# Identification and Characterization of 63 MicroRNAs in the Asian Seabass *Lates calcarifer*

**DOI:** 10.1371/journal.pone.0017537

**Published:** 2011-03-11

**Authors:** Jun Hong Xia, Xiao Ping He, Zhi Yi Bai, Gen Hua Yue

**Affiliations:** Molecular Population Genetics Group, Temasek Life Sciences Laboratory, National University of Singapore, Singapore, Republic of Singapore; East Carolina University, United States of America

## Abstract

**Background:**

MicroRNAs (miRNAs) play an important role in the regulation of many fundamental biological processes. So far miRNAs have been only identified in a few fish species, although there are over 30,000 fish species living under different environmental conditions on the earth. Here, we described an approach to identify conserved miRNAs and characterized their expression patterns in different tissues for the first time in a food fish species Asian seabass (*Lates calcarifer*).

**Methodology/Principal Findings:**

By combining a bioinformatics analysis with an approach of homolog-based PCR amplification and sequencing, 63 novel miRNAs belonging to 29 conserved miRNA families were identified. Of which, 59 miRNAs were conserved across 10–86 species (E value≤10^−4^) and 4 miRNAs were conserved only in fish species. qRT-PCR analysis showed that miR-29, miR-103, miR-125 and several let-7 family members were strongly and ubiquitously expressed in all tissues tested. Interestingly, miR-1, miR-21, miR-183, miR-184 and miR-192 showed highly conserved tissue-specific expression patterns. Exposure of the Asian seabass to lipopolysaccharide (LPS) resulted in up-regulation of over 50% of the identified miRNAs in spleen suggesting the importance of the miRNAs in acute inflammatory immune responses.

**Conclusions/Significance:**

The approach used in this study is highly effective for identification of conserved miRNAs. The identification of 63 miRNAs and determination of the spatial expression patterns of these miRNAs are valuable resources for further studies on post-transcriptional gene regulation in Asian seabass and other fish species. Further identification of the target genes of these miRNAs would shed new light on their regulatory roles of microRNAs in fish.

## Introduction

MicroRNAs (miRNAs) are short, endogenous noncoding RNAs (∼22 nucleotides in length) found in animals, plants and virus [1], [2], [3]. miRNA expression can be regulated at transcriptional and post-transcriptional levels during RNA biogenesis [4]. Recent studies showed that miRNAs might have potentially enormous importance in the regulation of many fundamental biological processes, such as cardiogenesis and myogenesis [5], [6], [7], neurogenesis [8], [9], hematopoiesis [10], stem cells, regeneration and homeostasis [11], [12], [13], [14], proliferation, differentiation, cell fate determination, apoptosis, signal transduction, organ development [15], [16], [17], [18], [19], [20], [21], immunological and inflammatory disorders [22], [23], [24]. The down-regulation of the expression of specific mRNA targets by miRNAs accounted for the approximately 70% detectable changes at the mRNA levels of all regulated proteins, either by directing the cleavage of mRNAs or interfering with translation [2], [25], [26]. miRNAs make up 1–5% of all genes making them the most abundant class of regulators in genome [27], [28].

Several approaches have been used to identify miRNAs. The first approach was through forward genetics such as the discovery of lin-4 in *Caenorhabditis elegans* [29], [30]. The second was directional cloning and sequencing by constructing a cDNA library, which has been used in *Arabidopsis thaliana* [31], rice *Oryza sativa* [32], rainbow trout *Oncorhynchus mykiss* [33] and zebrafish *Danio rerio* [34]. However, miRNAs expressing at a low level or only in a specific condition or specific cell types would be difficult to find with this approach [4]. Recently high-throughput sequencing strategies had greatly expanded the depth of small RNA cloning coverage [35], [36]. However, the sequencing data from these strategies were not yet saturated, as reflected by many of the new identified miRNAs that were represented only once in the sequence database. Furthermore, since some libraries were derived from a limited amount of tissues, some miRNAs that were only expressed in specific adult tissues were not available [34], [37]. Bioinformatics prediction was genome-wide and sequence-based computational predictions. This method was based largely on the phylogenetic conservation and the structural characteristics of miRNA precursors (pre-miRNA) [38] and/or known miRNA genes [39], [40], enabling one to overcome the problem in directly cloning. On the other hand, the process required knowledge of the complete genome sequence, species-specific miRNAs could not be accurately identified without this information [37] and bioinformatically predicted miRNAs should be validated for their expression by northern blotting or sequencing. Over the past few years, thousands of miRNAs had been identified. With release 14, the miRBase sequence database had broken through the 10000 entries (http://www.mirbase.org/). Further studies showed that most of the miRNA sequences and the stems of miRNA hairpins were highly conserved across species although a number of lineage-specific miRNAs and species-specific miRNAs had been identified [39], [41], [42], [43]. These studies suggested that the cloning of miRNAs for a particular species was plausible based on the conserved homologs among pre-miRNAs and/or mature miRNAs originating from related species.

There are over 30,000 fish species on the earth, representing 50% of all vertebrates [44]. Currently, the cloning and characterization of miRNAs from fishes have been carried out only for two model fish species (medaka *Oryzias latipes* [45] and zebrafish [34]) and one food fish species, rainbow trout [33]. The Asian seabass *Lates calcarifer* belonging to the family Latidae of the order Perciformes, is an important food resource in Southeast Asia. As a food fish species, the Asian seabass could be an excellent model organism for aquaculture-genomic studies due to its compact genome (700 Mb) and extremely high fecundity (1.7 million eggs/spawning) [46]. Therefore, the Asian seabass might offer a good system for understanding of fish biology, such as in infectious diseases, growth and organ development under aquatic environment. Recently massive mortalities of seabass caused by bacterial or viral infections had caused seriously economic losses [47], [48], [49]. Unfortunately, an effective approach to protect the fish from infections is still not developed to date. miRNAs might potentially have an enormous impact in the regulation of immunological and inflammatory disorders as well as growth development [16], [22], [23], [24]. Identification of miRNAs and their target genes was an important step toward understanding the regulatory networks, gene silencing mechanisms and for practical use for gene manipulations in the Asian seabass. To date, no miRNA is available for the Asian seabass. Conserved miRNAs likely played an important role in regulating basic cellular and developmental pathways from lower to higher organisms [41]. Nevertheless, in comparison to model fish species with a fully sequenced genome, e.g., zebrafish, the in silico identification of conserved miRNAs families in the Asian seabass was not possible due to the absence of whole genomic information for the species.

We attempted to clone and identify conserved miRNAs from the Asian seabass as the first step toward understanding the regulatory roles of miRNAs in fish living under different environmental conditions. To identify conserved miRNAs, bioinformatics analysis of the conservation and structural similarity of pre-miRNA sequences across related model species was performed first. Then an approach of homolog-based PCR amplification and sequencing were carried out. In this study, 63 novel miRNAs in the Asian seabass were identified. The approach developed in this study was highly effective for identification of conserved miRNAs. Quantitative real-time RT-PCR (qRT-PCR) revealed differential expressions of these miRNAs in 8 organs (gill, brain, eye, muscle, liver, intestine, heart and kidney) and in spleen of the Asian seabass before and post a challenge with lipopolysaccharide (LPS). Our data supply the basis for the understanding of the functions of miRNAs in fish.

## Results and Discussion

### 1. Identification of pre-miRNAs and mature miRNAs in the Asian seabass

To find conserved homlogs among fish species 623 pre-miRNA sequences from zebrafish (360), Fugu, *Fugu rubripes* (131) and *Tetraodon nigroviridis* (132) were retrieved from miRBase (the microRNA database; www.mirbase.org/). The conserved homologs of the pre-miRNAs were investigated using commercial software Sequencher. One hundred and six pairs of primers (Table S1) were designed based on the conserved fragments among species. For cloning less conserved pre-miRNAs, additional 47 pairs of primers were designed only based on the zebrafish pre-miRNA sequences since no countparts were found in Fugu and Tetraodon releases (Table S1).

Based on a Switching Mechanism At 5′ end of RNA Transcript (SMART) -based method for cDNA library construction [50], one full-length cDNA library using pooled mRNAs from 9 tissues was constructed and used as template in following PCR reactions for amplification of putative pre-miRNAs. This library provided a good tool to identify miRNAs differentially expressed in these tissues. Eight hundred clones were sequenced and 786 reads were obtained after trimming of end and vector sequences and screening to eliminate sequences with low quality. These sequences were further clustered to identify unique sequences. Finally 322 unique sequences (Table S2) were obtained.

**Figure 1:**
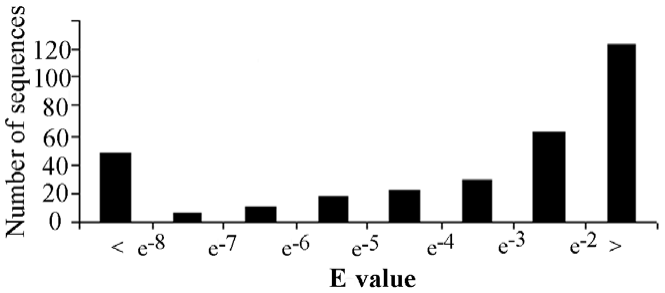
The distribution of the seabass miRNA precursor candidates in various E value intervals as identified by the BLASTn program showing conservation with known pre-miRNAs. Conservation of the 322 unique sequences that cloned from the seabass cDNA library with known pre-miRNAs was identified by the BLASTn program (E value). The most common conservation intervals were <e^−2^ (123 sequences), between e^−2^ and e^−3^ (63 sequences) and followed by <e^−8^ (48 sequences).

**Figure 2:**
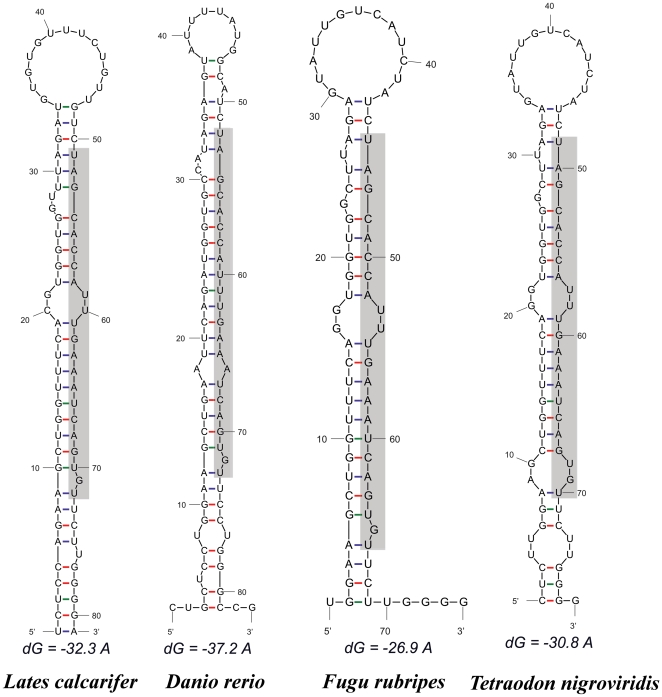
Conservation of the miR-29 family in Pisces. Predicted fold-back structures of mir-29 precursors from *Lates calcarifer*, *Danio rerio*, *Fugu rubripes*, *Tetraodon nigroviridis* and the folding energy (dG) for each fold-back structure are presented. The shadow in each precursor structure indicates the mature miR-29 sequence in the species.

Sequence analyses using the BLASTn program revealed that 107 of the unique sequences showed high conservation (E value≤e^−4^) with known pre-miRNAs (Table S2). By plotting blasted E value of the small RNAs against total number of unique sequences (Figure 1), we found that the most common conservation intervals were <e^−2^ (123 sequences), between e^−2^ and e^−3^ (63 sequences) and followed by <e^−8^ (48 sequences). Prediction of fold-back structures and energies performed with the DINAMelt server indicated that the stem-loop structure for many sequences were stable (with low folding energy) (Table S2) and lacked large internal loops or bulges. This is suggestive of the general characteristics of pre-miRNAs. One of the examples is shown in Figure 2. However, most of the unique sequences could not form a good stem-loop structure which might result from sequence artifacts (PCR errors) due to the formation of heteroduplex molecules, the error of Taq DNA polymerase [51] and/or nature of sequences since only partials of the pre-miRNAs were obtained in this study.

The unique sequences considered as putative seabass pre-miRNAs were then searched against the miRBase database to predict conserved mature miRNAs in the Asian seabass (designated as lcal-miRNAs). Based on the phylogenetic conservation with known pre-miRNAs and miRNAs and their stability of folding stem-loop structures, 108 sequences [64 unique sequences with high conservation (E value≤e^−4^) and 44 with less conservation (E value>e^−4^)] were selected to predict conserved mature miRNAs in the Asian seabass. These miRNAs might represent two classes of conserved miRNAs, i.e., miRNAs in broad conservation families and poor conservation families. Fold-back structures could be predicted for some of the putative pre-miRNAs in the Asian seabass.

To identify the nucleotides at the very 5′ and 3′ ends of the putative miRNAs by PCR, an adaptor-ligated miRNA cDNA library was constructed and used as template. The miRNA sequence-specific primers and universal primers (Table S1)were used in following PCR reactions. Five hundreds of clones were sequenced. Finally, the nucleotides at the very 5′ or 3′ ends were validated for 151 putative conserved mature miRNAs. Of which, 112 miRNAs with the very 3′ end and 102 miRNAs with the very 5′ end were identified. Upon combined the data, 63 miRNAs with the nucleotides at both of the very ends were confirmed, representing novel mature miRNAs identified in the Asian seabass (Table S3). The sizes of the 63 miRNAs were ranged from 19 to 25 bases. Of the 63 newly identified miRNAs, 48 began with a 5′ uridine, which was a characteristic feature of miRNAs, 11 began with a 5′ A, 2 began with a 5′ C and G. Names of the seabass miRNAs were assigned based on the homologies between the miRNA and published miRNA sequences in the Sanger database; the isoforms from one family were labeled in alphabetical order (Table S3).

### 2. Conservation and evolution of the identified miRNAs

Many miRNAs were highly conserved among organisms [52]. For example, at least a third of *C. elegans* miRNAs had homologs in humans [53]. The conservation among species suggests that miRNAs represent a relatively old and important regulatory pathway [54]. miRNAs conservation could be used to identify the novel miRNAs for species without reference genome sequences. Based on the sequence conservation between the lcal-miRNAs and published miRNA sequences in Sanger database, the 63 novel lcal-miRNAs were classified into 29 conserved miRNA families with a range of 1 to 15 loci per family (Table S4). Since the mature miRNA sequences from one family were highly conserved, the PCR product amplified with one primer pair possibly contained several similar miRNAs from one family. We identified 15 members of let-7 family and 4 members of miR-124 family in the Asian seabass. To date, 18 let-7 isoforms were identified in the zebrafish, and 10 let-7 isoforms for the Tetraodon and the Fugu respectively according to the miRBase database (release 14). Additionally, six miR-124 isoforms in zebrafish and three isoforms in Tetraodon and Fugu respectively were registered in the miRBase. The identified numbers for these two miRNA families in this study were comparable to the numbers in model species. In animals and plants, miRNAs exist as multigene families [37], therefore, it was not surprising to get so many isoforms for these families in the study.

**Figure 3:**
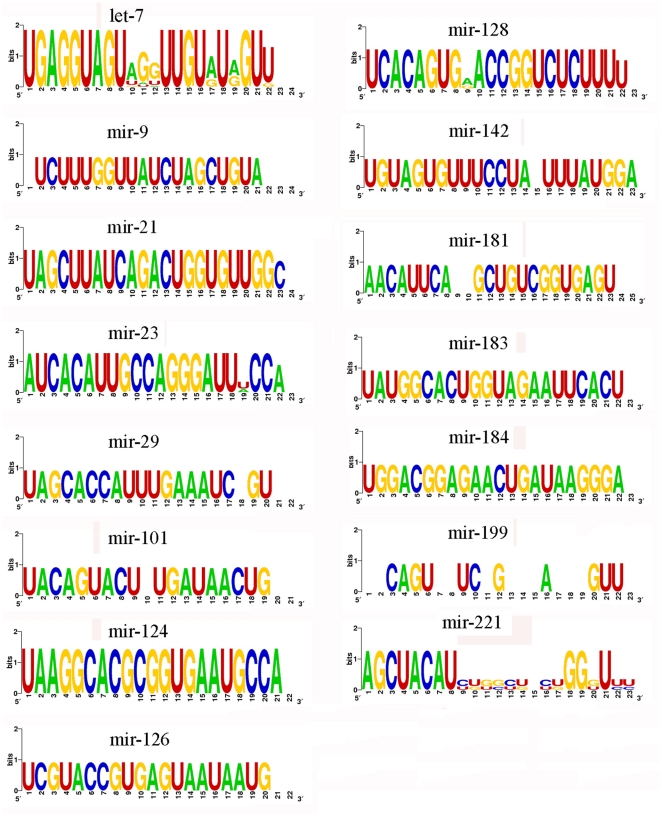
Sequence logos showing the most conserved bases for miRNAs in one family. The miRNA sequence logos for 15 lcal-miRNA families with multiple sequences (≥2) are presented. Each logo consists of stacks of symbols, one stack for each position in the sequence. The overall height of the stack indicates the sequence conservation at that position, while the height of symbols within the stack indicates the relative frequency of each nucleotide at that position. Some miRNAs from one family differ only by a few base pairs on either side and/or in the middle (e.g. let-7, mir-9, mir-21, mir-124 and mir-126); the mir-199 family is highly divergent, since the two miRNAs in that might be from the 5′ arm and from the 3′ arm of a pre-miRNA, respectively.

A sequence logo in bioinformatics was a graphical representation of the sequence conservation of nucleotides [55]. The sequence logos for 15 lcal-miRNA families with multiple miRNA sequences (≥2) were presented in Figure 3. Some of the sequences were found to have slight shifts in their 5′ and 3′ ends, such as family mir-21, mir-124, mir-126, mir-183, and mir-184, which is a common phenomenon in miRNA cloning and could be attributed to processing shifts or enzymatic modifications of miRNAs such as RNA editing, 3′ nucleotide additions or sequencing artifacts [37]. Some miRNAs from one family differed only by a few base pairs not only on either side, but also in the middle region, e.g., let-7, mir-23, mir-29, mir-101 and mir-128. The two members in mir-199 family were highly divergent, since the two miRNAs might be produced from the 5′ arm and the 3′ arm of a pre-miRNA, respectively. Sequence logos showed most of the bases for miRNAs in one family were highly conserved.

**Figure 4:**
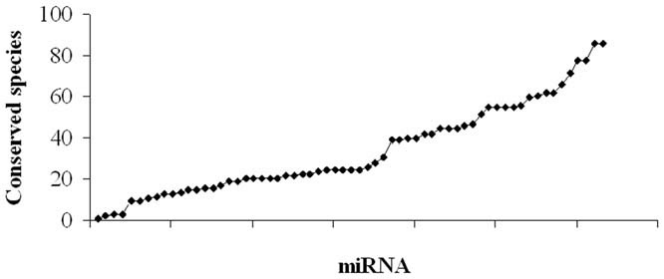
Conservation of the seabass mature miRNAs across species. The 63 miRNAs validated by PCR and sequencing for the Asian seabass were searched against the miRBase database to identify the conservation across species. Of which, 59 miRNAs were conserved across 10–86 species and 4 miRNAs were found to be conserved less than 4 species according to our conservation criteria (E value≤10^−4^). Each small black rhombus represents an identified miRNA.

Sequence similarity searches against the central miRNA registry also showed that most of the miRNAs were conserved across many species (Figure 4). Of which, 59 miRNAs were conserved across 10–86 species according to our conservation criteria (a blast E value≤10^−4^ for mature miRNA and precursor). For example, lcal-miR-20 was conserved in all lineages of Vertebrata, including Amphibia, Mammalia, Pisces and Aves. lcal-miR-29a,b and many members of the lcal-let-7 family were conserved in Bilateria, including Deuterostoma, Lophotrochozoa and Ecdysozoa. High conservation of the miRNA families suggested an evolutionary conserved function.

A number of lineage-specific miRNAs and species-specific miRNAs were discovered recently. For example, many miRNAs that were recently discovered in human and chimpanzee were not conserved beyond mammals, and ∼10% were taxon-specific [39]. In our study we have also cloned some miRNAs that only conserved among closely related species. For example, 4 miRNAs (lcal-let-7l, lcal-miR-21a, lcal-miR-101b and lcal-miR-724a) were conserved only in fish species (E value≤10^−4^), indicating these miRNAs were fish-specific and might play a key role in the evolutionary process of fish.

### 3. Expression patterns of the Asian seabass miRNAs

**Figure 5:**
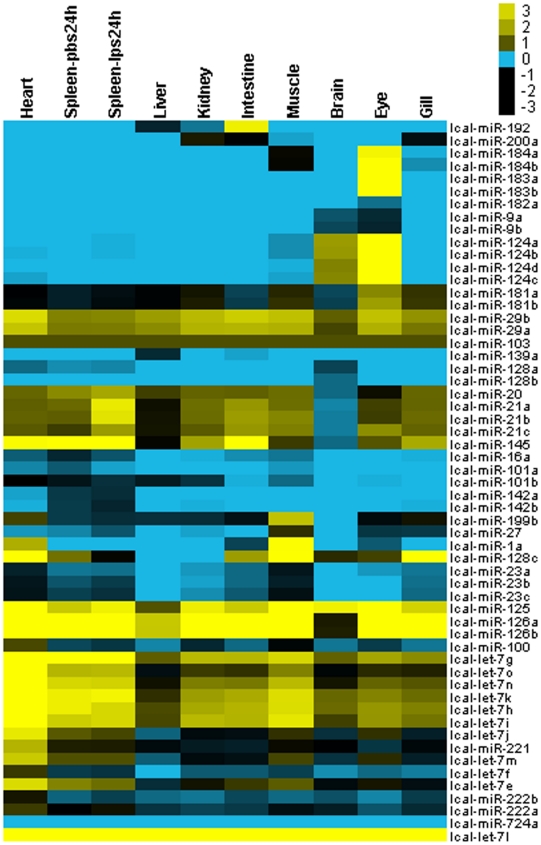
Expressions of the 57 conserved mature miRNAs in eight organs of the Asian seabass. The expression of 63 miRNA was determined in 8 organs (gill, brain, eye, muscle, liver, intestine, heart and kidney) and in spleen sampled at 24 hour post challenge with LPS and control samples by real-time PCR. Six (lcal-miR-199a, lcal-miR-152, lcal-let-7a,b,c,d) of the sequencing validated miRNAs without positive results in all of the evaluated tissues were deleted from the data. Data were presented on a logarithmic scale. The relative expression of each gene shown in the figure was the average of triplicate real-time PCR reactions, normalized to lcal-miR-103 gene expression. Yellow shading indicated increased levels of expression, and gray shading represented decreased levels of expression relative to the center. Blue color denoted undetectable expression in tissues.

Information about the expression of a miRNA is useful for the understanding of its functions [56]. The expression of miRNAs was tightly regulated both in time and space [37]. Hence, to validate expression and assist with the determination of functions, the expression for the 63 miRNAs in 8 different organs (gill, brain, eye, muscle, liver, intestine, heart and kidney) and in spleen following 24 h post-challenge with LPS and control sample were examined by qRT-PCR (Figure 5). Each reaction was performed in triplicate. We selected lcal-miR103 as the reference gene in the analysis as suggested in Peltier et al. [57]. Six (lcal-miR-199a, lcal-miR-152, lcal-let-7a, b, c and d) of sequencing validated miRNAs did not provide positive results in all of the evaluated tissues. These might represent miRNAs with low abundance or expressed only in few cells or tissues or resulted from the low quality of primers used. Based on miRNA abundance, hierarchical clustering analysis showed that the expression profiles between brain and eye and between kidney and intestine were more similar (Figure 5).

Many of the conserved miRNAs were expressed ubiquitously in rainbow trout [33]. In the Asian seabass, miR-29, miR-103, miR-125 and several let-7 family members were strongly and ubiquitously expressed in all tissues tested (Figure 5). Our results suggest these miRNAs might play an important role in the regulation of constitutive processes in diverse tissues. However, miRNAs were also expressed in a tissue-specific manner which provided clues about their physiological functions [2]. For example, in zebrafish, many miRNAs were highly expressed at later stages of development [34], [58]. In our study many miRNAs showed highly conserved tissue-specific expression patterns (Figure 5). For example, interesting expression was observed for miR-183-1 and miR-183-2, which were highly specifically expressed in eye and barely detectable in remaining tissues. Some studies demonstrated that miR-183 family members were expressed abundantly in specific sensory cell types in the eye, nose, and inner ear and contributed specifically to neurosensory development or function [59], [60]. Previous studies had shown that miR-1 was one of the highly conserved miRNAs and was abundantly and specifically expressed in the heart and other muscular tissues in fish [5], [27], [61], [62], *C. elegans* [63] and mouse [64]. Our study also showed that miR-1 was strongly expressed in muscle and heart, and weakly expressed in eye and intestine of the Asian seabass. Like other studies, our study suggests that miR-1 play an important role in regulation of muscle gene expression in the Asian seabass. In addition, miR-192 was highly expressed in intestine but moderate in liver and kidney. miR-184-1 and miR-184-2 were strongly expressed in eye and moderately expressed in muscle. Our expression data provide a basis for further understanding of regulatory roles of miRNAs in fish living under different aquatic conditions.

### 4. Potential functions of the identified miRNAs in an acute inflammatory response induced by LPS and vibrio bacteria

Our previous study had shown that exposure of Asian seabass at the age of 35 days post hatchery (dph) to LPS led to a dramatic increase of the expression of 25 immune-related genes in spleen by inducing an acute inflammatory response at 24 h post challenge [65]. This study showed that 14 of the 63 miRNAs were found to express at a very low levels (<1/100 fold of the reference gene; Figure 5) in spleen. Examination of the mean expression changes among the rest 49 miRNAs showed that exposure to LPS resulted in a general elevation in mature miRNA levels. A similar result was found in Moschos et al. [66]. Following LPS challenge, they observed rapid and transient increase in both the mean (4.3-fold) and individual levels of miRNA expression (46 of 104 miRNAs) in mouse lung. In this study, the expression for 34 of the 63 miRNAs (54%) was increased and only 12 of the 63 miRNA genes (19%) were down-regulated in spleen of the Asian seabass. Highly differentiated expression (>1.5fold) was summarized in Table 1. The miR-21 was highly up-regulated and miR-101 was highly down-regulated after exposure to LPS.

**Table 1:** Differential expression (>1.5 fold) of lcal-miRNAs in spleen of the Asian seabass at 24 h post challenge with LPS as revealed by real-time PCR.

miRNA expression	Folds of differential expression			
	1.5–2.0 fold	2.0–2.5 fold	2.5–3.0 fold	>3.0 fold
Up regulated	lcal-miR-192		lcal-miR-21c	lcal-miR-21a
	lcal-miR-27			lcal-miR-21b
	lcal-miR-124a			
	lcal-miR-124b			
	lcal-miR-124c			
	lcal-miR-124d			
	lcal-miR-222a			
Down regulated	lcal-miR-199b	lcal-miR-101a		
	lcal-miR-16a			
	lcal-miR-9a			
	lcal-miR-9b			
	lcal-miR-128c			

Vibrio infection of fish can cause significant mortality in fish mariculture, e.g., seabass [67]. To explore the function of identified miRNAs in an acute inflammatory response caused by vibrio bacteria, the temporal expression patterns of lcal-miR-21a in three immune-related organs, the spleen, kidney and liver of *Vibrio harveyi*-challenged seabass were examined by qRT-PCR. Interleukin-1 beta (IL-1β) is an important mediator of the inflammatory response [68], [69]. It was evident that the high level expression of IL-1β was caused by the presence of the pathogen in kidney (179 fold increased) at 1–3 hours post injection (hpi) and in spleen (234 fold increased) and liver (143 fold increased) at 6 hpi (Figure S1). This data indicated that an acute inflammatory response was induced in the challenged fish within 1 hpi. Further study by qRT-PCR analysis indicated that the miR-21 gene was also remarkably elevated in kidney (1.71 fold) at 3 hpi, spleen (2.21 fold) at 12 hpi and liver (4.65 fold) at 24 hpi (Figure S2).

The miR-21, being one of the most abundant miRNAs, was functioned as an anti-apoptotic factor and oncogene related to cell growth [33], [70], [71], [72], [73], [74]. In our study the miR-21 was highly expressed in several tissues and highly up-regulated in spleen at 24 hour post challenge by LPS and in three immune-related organs of *Vibrio harveyi*-challenged seabass. These results were consistent with the findings of previous studies [72], [73], [74], [75] demonstrating the importance of the miRNAs in acute inflammatory immune responses with protection against pathogen.

### Conclusions

By combining a bioinformatics analysis with an approach of homolog-based PCR amplification and sequencing, 107 unique sequences showing high conservation with known pre-miRNAs were obtained; and 63 novel miRNAs belonging to 29 conserved miRNA families were identified for the first time in the Asian seabass. The methods used in this study were effective in identifying a large number of highly conserved miRNAs as well as less conserved miRNAs, and could be applied to identify conserved miRNAs expressed at a low level that were difficult to clone by traditional methods in other fish species.

The pre-miRNAs cloned in this study provide the basis for future cloning of primary miRNAs and conducting functional analysis. The determination of the spatial expression patterns of these miRNAs is a valuable resource for further study on post-transcriptional gene regulation in Asian seabass and other fish species. Further identification of the target genes of these miRNAs could shed new light on their regulatory roles of miRNAs in fish.

## Materials and Methods

### Ethics statement

All handling of fishes was conducted in accordance with the guidelines on the care and use of animals for scientific purposes set up by the Institutional Animal Care and Use Committee (IACUC) of the Temasek Life Sciences Laboratory, Singapore.

### Fish, LPS and *Vibrio harveyi* challenge and sampling

One hundred of individuals of small Asian seabass (15 dph) were transported from a commercial fish farm to the animal house at the Temasek Life Sciences Laboratory. The fishes were maintained in a large tank containing 500 L seawater at 25°C for three weeks of acclimation. Fishes were fed twice daily with pelleted feed (Zhongshan Tongyi, Taiwan). One day prior to challenge, 16 of healthy fishes of average weight 5 g were transferred to two smaller tanks holding 10 L of seawater. For 8 fishes in tank 1 each fish were injected intraperitoneally with 0.1 ml of 2 mg/ml of *Escherichia coli* LPS (Sigma-Aldrich, MO, USA) by dilution with phosphate buffered saline at room temperature. In tank 2 (control), a total of 8 fishes was received an intraperitoneal injection of 0.1 ml of phosphate buffered saline for each fish. Just before injection and sampling, the fishes were anaesthetized using AQUI-S® with a concentration of 15 mg/L (AQUI-S New Zealand Ltd, Lower Hutt, New Zealand). Eight fishes from each tank were sacrificed at 24 h post challenge. Spleen was taken for each fish from each tank and kept in Trizol reagent (Invitrogen, CA, USA) at −80°C until use. In addition, tissues including gill, brain, eye, muscle, liver, intestine, heart, spleen and kidney of 5 untreated seabass fishes were also taken and kept at −80°C.

To explore the temporal expression patterns of lcal-miR-21a in three immune-related organs, thirty Asian seabass (at the age of three months) were transferred to two tanks holding 200 L of seawater. For 15 fishes in test tank each fish were injected intraperitoneally with 0.1 ml of phosphate buffered saline dissolved culture pellet of *Vibrio harveyi* (∼e10 copy/ml) at room temperature. In control tank, a total of 15 fishes was received an intraperitoneal injection of 0.1 ml of phosphate buffered saline for each fish. Just before injection and sampling, the fishes were anaesthetized using AQUI-S® with a concentration of 15 mg/L (AQUI-S New Zealand Ltd, Lower Hutt, New Zealand). Three fishes from each tank were sacrificed at 1, 3, 6, 12, 24 hpi. Spleen, kidney and liver were taken for each fish from each tank and kept in Trizol reagent (Invitrogen, CA, USA) at −80°C until use.

### Construction of a full-length cDNA library and a miRNA library

Total RNA was isolated separately from gill, brain, eye, muscle, liver, intestine, heart, spleen and kidney of the Asian seabass using TRIzol (Invitrogen, CA, USA) according to the manufacturer's instructions. Purification of mRNA from total RNA was carried out using Oligotex mRNA Midi Kit (Qiagen, CA, USA). The resulting mRNA from 9 tissues of 5 fishes was mixed with equal quantity separately. One µg of the mixed mRNAs were used for cDNA library construction using a SMART-based method. The reverse transcription was carried out with PowerScript reverse transcriptase following the manufacturer's protocol (Clontech, CA, USA). A oligo(dT) primer [CDS III/3′ PCR Primer 5′-AAG CAG TGG TAT CAA CGC AGA GTA C(T)_30-_3′] was used to prime the first-strand synthesis reaction, and the SMART IV Oligo (5′-AAG CAG TGG TAT CAA CGC AGA GTA CGC rGrGrG-3′) served as a short, extended template at the 5′ end of the mRNA. The resulting single strand cDNAs serving as a template was amplified by SMART-Oligo-IIA-primer (5′-AAG CAG TGG TAT CAA CGC AGA GT-3′) and the PCR products were kept at −80°C till use.

A miRNA library was constructed as described [76] with slight modification. In brief, enrichment of small RNAs from total RNA was performed with mirVana™ miRNA isolation kit (Ambion, CA, USA) following the instruction manual and then was separated on a denaturing 15% polyacrylamide gel. The nucleotides from positions 18–25 bp were size fractionated. RNA was eluted overnight with 0.4 M NaCl at 4°C and recovered by ethanol precipitation with glycogen. The purified small RNAs were then ligated to a 3′ adaptor [5′-(Pu)uu AAC CGC GAA TTC CAG (idT)-3′; where lowercase letters indicate RNA, uppercase letters indicate DNA, Pu denotes 5′-phosphorylated uridine, and idT represents 3′-inverted deoxythymidine.] and a 5′ adaptor (5′-GAC CAC GCG TAT CGG GCA CCA CGT ATG CTA TCG ATC GTG AGA TGG G-3′). Reverse transcription was performed with PowerScript reverse transcriptase (Clontech, CA, USA) and RT primer (5′-GAC TAG CTG GAA TTC GCG GTT AAA-3′). The resulting 1^st^ strand cDNA was kept at −80°C till use.

### Design of degenerate primers and amplification of pre-miRNA sequences

Pre-miRNA sequences of zebrafish (360), fugu (131) and Tetraodon (132) were retrieved from miRBase (the microRNA database release 14; http://www.mirbase.org/). All of the sequences were aligned with software Sequencher 4.9 (Gene Codes, MI, USA). The conserved fragments were used to design degenerate primers with the EditSeq program in DNAstar 7 (DNASTAR, WI, USA). The melting temperatures for these primers were designed at around 60°C.

For amplification of pre-miRNA sequences 153 pairs of degenerate primers (Table S1) were used. Single strand cDNA diluted 20 times was used as DNA template for PCR. The resulting PCR products were directly inserted into a pGEM-T vector (Promega, WI, USA) and transformed into *E. coli* strain XL-1 (Stratagene, CA, USA). For each product 4–6 clones were sequenced using BigDye chemicals and ABI 3730xl Genetic Analyzer (Applied Biosystems, CA, USA).

### Prediction and determination of putative seabass pre-miRNAs

Trimming of vector sequences and low-quality regions from source sequences was performed using commercial software Sequencher 4.9 (Gene Codes, MI, USA). All trimmed sequences were used to form contigs. Singletons and consensus sequences of each contig were referred as unique sequences and were used to search the miRBase (the microRNA database release 14; http://www.mirbase.org/ ) [77], [78], [79] with parameters (BLASTn and stem-loop sequences) to find conservation of putative pre-miRNA sequences. Prediction of fold-back structures and energies were performed with the DINAMelt server (Prediction of Melting Profiles for Nucleic Acids; http://frontend.bioinfo.rpi.edu/applications/hybrid/quikfold.php) and with RNA 3.0 as energy rule. Based on these analyses, sequences with low folding energy (stable stem-loop structure) and conservation with known pre-miRNAs were considered as putative seabass pre-miRNAs and were then searched against the miRBase database (release 14) with parameters (BLASTn and mature miRNAs sequences) to predict conserved mature miRNAs in Asian seabass (designated as lcal-miRNAs).

### Mapping boundary ends of putative miRNAs by PCR and sequencing

Sequence-specific primers with partial coverage of the putative miRNA sequences (Table S1) and general primers that matched to the adaptor sequences were used in the following PCR reactions. For mapping the 5′ end to the very nucleotide by PCR, gene-specific primers specific to the 3′ ends of putative miRNAs and a mRAP-5′ PCR primer (5′-GCG TAT CGG GCA CCA CGT ATG C-3′) were used; for mapping the 3′ end to the very nucleotide by PCR, gene specific primers specific to the 5′ ends of putative miRNAs and a mRAP-3′ PCR primer (5′-GAC TAG CTT GGT GCC GAA TTC GCG GTT AAA-3′) were used. Briefly, 25 µl of reaction including 0.5 U HotStar Taq DNA Polymerase (Qiagen, CA, USA), 1×PCR Buffer, 100 µm dNTPs, 200 nmol of each primer and 1 µl diluted 1^st^ strand miRNA cDNA solution was initially denaturated at 95°C for 15 min, then amplified for 35 cycles (95°C, 30 s, 60°C, 30 s and 72°C, 20 s). The PCR products were separated on a 10% polyacrylamide gel. The fragments were size fractionated, purified and inserted into a pGEM-T vector (Promega, WI, USA) for sequencing. Automated base calling of the raw sequences for mapping of boundary ends of putative conserved miRNAs and removal of vector and adaptor sequences were performed with commercial software Sequencher 4.9. All trimmed sequences were used to search against the miRBase database (release 14) with BLASTn to find the conservation of putative miRNA sequences. Sequences between 19 and 25 bp in length with a blast E value<e^−4^ were considered as miRNAs in Asian seabass. The miRNA sequence logos for 15 miRNA families with multiple miRNA sequences (≥2) were produced with program WebLogo Version 2.8 [55], [80].

### Analysis of expression of mature miRNAs through quantitative real-time RT-PCR (qRT-PCR) in Asian seabass

Gene-specific RT primers (Table S1) for 63 identified lcal-miRNAs were designed according to their mature miRNA sequences. Primers (lca-IL1b) for *IL-1β* gene were designed based on the seabass *IL-1β* EST sequence (Genbank acc. No. EX468370). The gene-specific RT primers were equally mixed used as RT primers for first strand cDNA synthesis. In brief, total RNA was isolated from gill, brain, eye, muscle, liver, intestine, heart and kidney of 3 seabass fishes and spleen from 3 LPS-challenged fishes and 3 PBS-treated fishes (control group) using TRIzol (Invitrogen, CA, USA). The concentration and purity of total RNA were examined using a NanoDrop ND-1000 Spectrophotometer (NanoDrop Technologies, NC, USA). Around 2 µg total RNA for each sample was treated with RNase-free DNase I (Promega, WI, USA) following the manufacturer's protocol. Reverse transcription was performed at 25°C for 10 minutes, then 42°C for 60 minutes with a final incubation at 75°C for 15 minutes using gene-specific RT primer cocktail listed in the supplementary material (Table S1), a 5′ adaptor (5′-GAC CAC GCG TAT CGG GCA CCA CGT ATG CTA TCG ATC GTG AGA TGG G-3′) and PowerScript reverse transcriptase (Clontech, DB, USA). The remaining reagents (buffer, dNTPs, dithiothreitol, RNase inhibitor, Thermoscript] were added as specified in the Thermoscript protocol. The reverse transcription of the tRNA from the spleen, kidney and liver of three *Vibrio harveyi*-challenged fishes and three control fishes sampled at each time point (1, 3, 6, 12, 24 hpi) were performed as above, but using a mixture of gene-specific RT primers and hexamer primers as RT primers for first strand cDNA synthesis.

Real-time quantitative PCR was performed with the iQ SYBR Green Supermix (Bio-Rad, CA, USA) as described by the manufacturer in an iQ™5 Real-Time PCR Detection Systems (Bio-Rad, CA, USA). PCR amplicons for gene-specific qRT-PCR primer pairs (Table S1) were validated by the presence of one peak as shown by the melting curve. The curve was generated by the thermal denaturing protocol that followed each real-time PCR run. Briefly, 25 µl of reaction including 12.5 µl SYBR Green Supermix, 200 nmol each primer and 1 µl 10-times-diluted 1^st^ strand cDNA solution was initially denaturated at 95°C for 3 min, then amplified for 40 cycles (95°C, 5 s, 60°C, 10 s and 72°C, 20 s). PCR was performed in triplicates. Values shown in Figure 5 were the average of triplicate real-time PCR reactions, normalized to lcal-miR-103 gene expression. Gene expression datasets were analyzed by Cluster 3.0 which was originally developed by Michael Eisen, with parameters as hierarchical clustering, uncentered correlation, complete linkage (http://bonsai.ims.u-tokyo.ac.jp/~mdehoon/software/cluster/software.htm#ctv), and visualized in software Java TreeView [81]. Values shown in Figure S1 and S2 were the average of triplicate real-time PCR reactions, normalized to the test gene expression in the control at the respective time points.

## Supporting Information

Figure S1 **IL-1ß expression pattern in three immune-related organs of Vibrio harveyi-challenged seabass at different time points.**
(TIF)

Figure S2 **Lcal-miR-21a expression pattern in three immune-related organs of Vibrio harveyi-challenged seabass at different time points.**
(TIF)

Table S1 **Primer sequences used for cloning and analyzing the expression of the Asian seabass miRNAs.**
(XLS)

Table S2 **The 322 unique sequences identified by the Sequencher assembly program and their blast results against the miRBase database.**
(XLS)

Table S3 **Novel miRNAs in the Asian seabass that are homologous to known miRNAs from other species.**
(XLS)

Table S4 **Classification of the 63 newly cloned miRNAs in the Asian seabass into miRNA families.**
(DOC)
