# Supplementary figures and images for: Identification and Characterization of 63 MicroRNAs in the Asian Seabass Lates calcarifer
Source: PLoS One. 2011 Mar 11;6(3):e17537. doi: 10.1371/journal.pone.0017537 (PMC3055879; doi:10.1371/journal.pone.0017537)

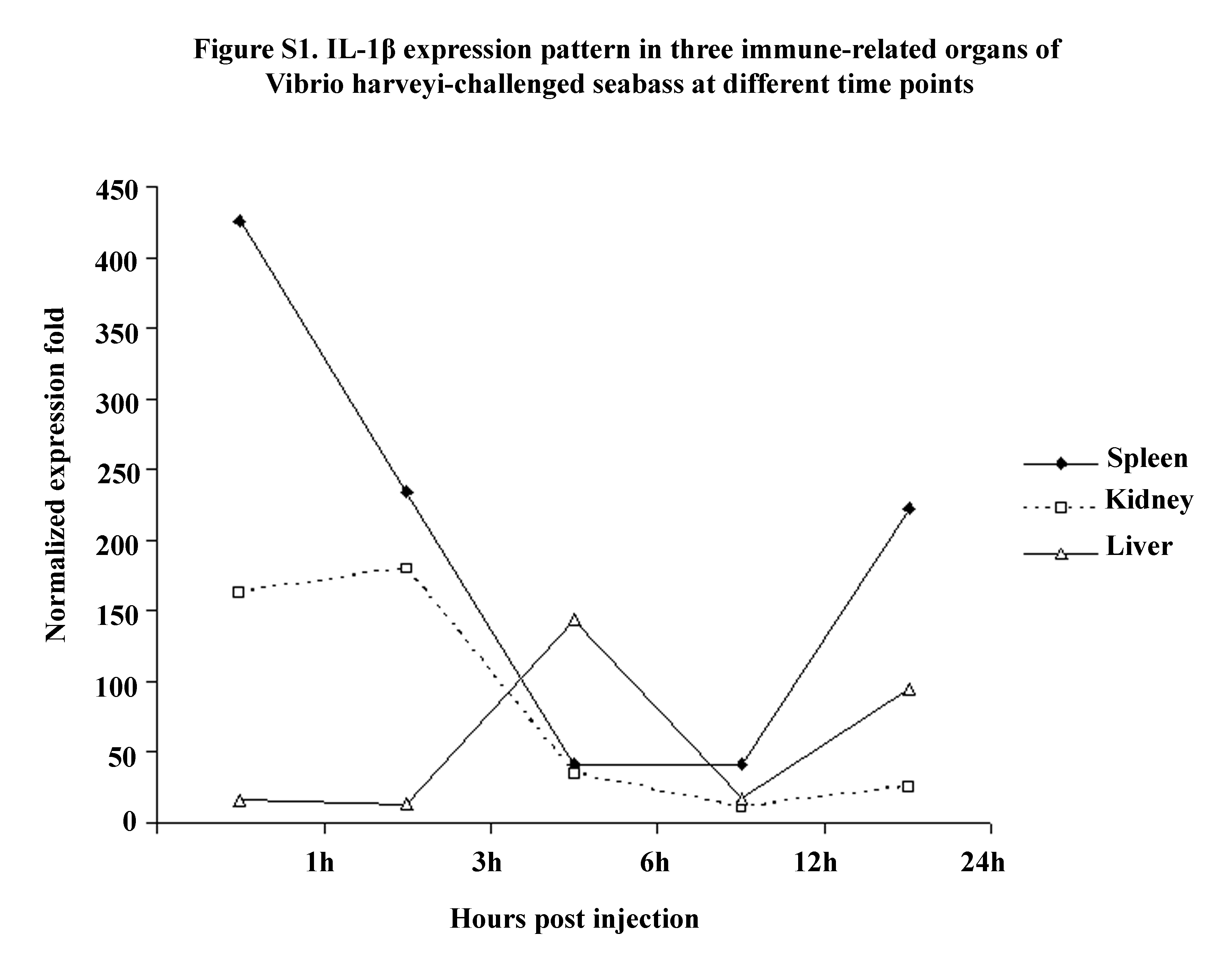

Supplement: Figure S1 — IL-1ß expression pattern in three immune-related organs of Vibrio harveyi-challenged seabass at different time points. (TIF) [file pone.0017537.s001.tif]

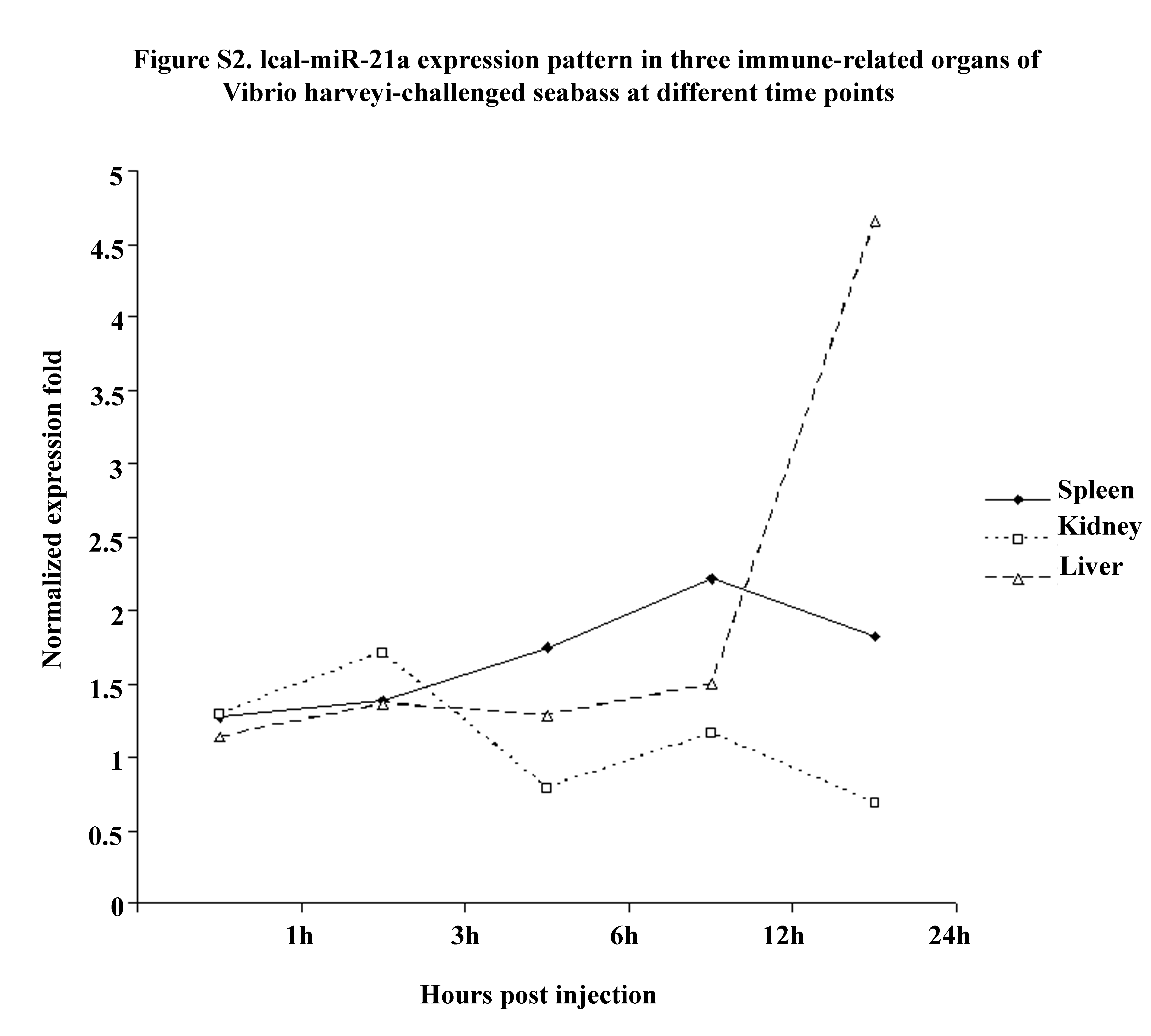

Supplement: Figure S2 — Lcal-miR-21a expression pattern in three immune-related organs of Vibrio harveyi-challenged seabass at different time points. (TIF) [file pone.0017537.s002.tif]
