# Supplementary material for: Identification and Characterization of 63 MicroRNAs in the Asian Seabass Lates calcarifer
Source: PLoS One. 2011 Mar 11;6(3):e17537. doi: 10.1371/journal.pone.0017537 (PMC3055879; doi:10.1371/journal.pone.0017537)
Supplement: Table S4 — Classification of the 63 newly cloned miRNAs in the Asian seabass into miRNA families. (DOC) [file pone.0017537.s006.doc]

**Table S4. Classification of the 63 newly cloned miRNAs in the Asian seabass into miRNA families**

| **Gene family** | **miRNA number** | **Gene family** | **miRNA number** |
| --- | --- | --- | --- |
| let-7 | 15 | mir-126 | 2 |
| mir-1 | 1 | mir-128 | 3 |
| mir-8 | 1 | mir-139 | 1 |
| mir-9 | 2 | mir-142 | 2 |
| mir-15 | 1 | mir-145 | 1 |
| mir-17 | 1 | mir-148 | 1 |
| mir-21 | 3 | mir-181 | 2 |
| mir-23 | 3 | mir-182 | 1 |
| mir-27 | 1 | mir-183 | 2 |
| mir-29 | 2 | mir-184 | 2 |
| mir-99 | 1 | mir-192 | 1 |
| mir-101 | 2 | mir-199 | 2 |
| mir-103 | 1 | mir-221 | 3 |
| mir-124 | 4 | mir-724* | 1 |
| mir-125 | 1 |  |  |
